# Editorial Comment: Intradetrusor botulinum toxin injections (300 units) for the treatment of poorly compliant bladders in patients with adult neurogenic lower urinary tract dysfunction

**DOI:** 10.1590/S1677-5538.IBJU.2022.01.05

**Published:** 2021-12-21

**Authors:** Marcio Augusto Averbeck

**Affiliations:** 1 Hospital Moinhos de Vento Unidade de Videourodinâmica Porto Alegre RS Brasil Chefe de Neuro-Urologia, Unidade de Videourodinâmica, Hospital Moinhos de Vento. Porto Alegre, RS, Brasil

R Corey O’Connor 1, Dane P Johnson 2, Michael L Guralnick 1

Neurourol Urodyn. 2020 Nov;39(8):2322-2328.

## COMMENT

Intradetrusor onabotulinum toxin A (BTX) is the most effective minimally invasive treatment to reduce neurogenic detrusor overactivity (DO) (1) and has been proven effective in patients with neurourological disorders in phase III randomized placebo-controlled trials (2,3). On-label dosage for neurogenic DO is 200 Units. Patients with spinal dysraphism and spinal cord injury (SCI) have a higher risk of developing upper urinary tract deterioration (UUTD). Urodynamic findings such as reduced compliance and high detrusor leak point pressure (DLPP) are major risk factors for UUTD (4). Additionally, reduce bladder compliance has been identified as a factor related to poor response to intradetrusor BTX.

O’Connor et al retrospectively evaluated patients with impaired bladder compliance (≤20 mL/cm H2O) secondary to spinal myelopathy treated with 300 units of intradetrusor onabotulinum toxin A (BTX). Inclusion criteria comprised patients ≥18 years of age with neurogenic lower urinary tract dysfunction with reduced compliance refractory to medical therapy treated with BTX injections. Objective improvement in compliance was defined as an increase ≥5 mL/cm H2O on repeat urodynamics (UDS). Seventy-one patients were included, 35 with myelomeningocele (MMC) and 36 with acquired spinal cord injuries (SCI). Mean age was 37.2 years (range: 18-78) and ANLUTD duration was 14.5 years (range: 1-34). Average pre-injection bladder compliance was 9.2 mL/cm H2O (range: 3.0-16.7). After treatment with BTX, 37 of 71 (52%) patients reported subjective reductions in lower urinary tract symptoms. BTX injections significantly improved bladder compliance in 31% and normalized storage pressures in 25% of neuro-urological patients with poorly compliant bladders refractory to medications. Individuals with shorter time intervals since neurologic injury responded better to BTX than those with longer durations (P=0.032).

These findings are clinically relevant since an increase (≥5 mL/cm H2O) in bladder compliance was identified in one-third of patients on repeat UDS after 300 units of BTX. Etiology of neurogenic lower urinary tract dysfunction not predictive of BTX success. One can hypothesize that patients with longstanding neurological diseases may have a greater degree of fibrosis within the bladder wall and, as a result, may be less likely to improve after BTX injections. However, further studies are still needed to identify which neuro-urological patients should receive a higher BTX dosage in the minimally-invasive treatment of neurogenic DO.
